# Blood Omega-3 Fatty Acids Are Inversely Associated With Albumin-Creatinine Ratio in Young and Healthy Adults (The Omega-Kid Study)

**DOI:** 10.3389/fcvm.2021.622619

**Published:** 2021-04-27

**Authors:** Mark G. Filipovic, Martin F. Reiner, Saskia Rittirsch, Irina Irincheeva, Stefanie Aeschbacher, Kirsten Grossmann, Martin Risch, Lorenz Risch, Andreas Limacher, David Conen, Juerg H. Beer

**Affiliations:** ^1^Institute of Anesthesiology, Cantonal Hospital of Winterthur, Winterthur, Switzerland; ^2^Department of Internal Medicine, Cantonal Hospital of Baden, Baden, Switzerland; ^3^Center for Molecular Cardiology University of Zurich, Zurich, Switzerland; ^4^Department of Internal Medicine, Cantonal Hospital of Baden, Baden, Switzerland; ^5^Clinical Trials Unit (CTU) Bern, University of Bern, Bern, Switzerland; ^6^Cardiovascular Research Institute Basel and Division of Cardiology, University Hospital Basel, University of Basel, Basel, Switzerland; ^7^Labormedizinisches Zentrum Dr Risch, Vaduz, Liechtenstein; ^8^Division of Laboratory Medicine, Cantonal Hospital Graubünden, Chur, Switzerland; ^9^Department of Laboratory Medicine, Institute of Clinical Chemistry, Inselspital Bern University Hospital, University of Bern, Bern, Switzerland; ^10^Private University Triesen, Triesen, Liechtenstein; ^11^Population Health Research Institute, McMaster University, Hamilton, ON, Canada

**Keywords:** omega-3-fatty acids, nutrition, prevention, population, kidney, albuminuria, albumin-creatinine ratio

## Abstract

**Background:** Omega-3 fatty acids are associated with a lower risk of cardiovascular disease (CVD) and with beneficial effects on CV risk factors. The albumin-creatinine ratio (ACR) is a risk factor for CVD, all-cause mortality and accelerated glomerular filtration rate (GFR) decline in the general population. We aimed to investigate the association between n-3 PUFAS and ACR in heathy individuals with preserved GFR.

**Design and Methods:** The present cross-sectional analysis is part of the GAPP study, a population-based cohort of healthy adults aged 25–41 years. Individuals with known CVD, diabetes, or a BMI >35 kg/m^2^ were excluded. eGFR was calculated according to the combined Creatinine/Cystatin C CKD-EPI formula. ACR was obtained from a fasting morning urine sample. The Omega-3 Index (relative amount of EPA and DHA of total fatty acids in %) was obtained from whole blood aliquots.

**Results:** Overall, 2001 participants (median age 37 years IQR 31; 40, 53% female) were included in this analysis. Median Omega-3 Index was 4.59 (IQR 4.06; 5.25) and median eGFR 111 ml/min/1.73 m^2^ (IQR 103; 118). Median ACR was 0.14 mg/mmol (IQR 0; 0.43). We found a significant inverse association of the Omega-3 Index with ACR (ratio 0.84, 95%CI 0.73–0.96; p = 0.011) which remained after comprehensive adjustment (ratio 0.86, 95%CI 0.74–1.00; *p* = 0.048). No association of the Omega-3 Index with eGFR was found. The adjusted difference in eGFR per 1-unit increase in Omega3-Index was −0.21 (95%CI −0.76; 0.35; *p* = 0.47).

**Conclusions:** A higher Omega-3 Index was significantly associated with lower ACR in this young and healthy population with preserved eGFR. Omega-3 fatty acids may exhibit cardio- and nephroprotective effects in healthy individuals through modulation of ACR.

## Introduction

Omega-3 polyunsaturated fatty acids (n-3 PUFAs) include the fish-derived eicosapentaenoic (EPA) and docosahexaenoic acid (DHA) as well as alpha-linolenic acid (ALA) derived from plants. Their beneficial effect on cardiovascular disease and risk factors has been the focus of a multitude of experimental studies and clinical investigations. Of note, they are associated with anti-inflammatory as well as antithrombotic activity (1), they are likely to improve endothelial dysfunction (2) and positively influence cardiovascular risk factors like HbA1c (3), triglycerides (3), and blood pressure (4).

Chronic kidney disease (CKD) and albuminuria as an early marker for kidney damage are both well-established cardiovascular risk factors (5, 6). In animal models dietary supplementation with n-3 PUFAs slows the progression of kidney disease and reduces urine albumin excretion as well as renal inflammation and fibrosis (7, 8). In older community-dwelling individuals, higher plasma PUFAs were associated with a lower age-related decline in kidney function (9). Data form interventional trials showed the potential of n-3 PUFA supplementation to attenuate the progression of albuminuria in individuals with type 2 diabetes and coronary heart disease (10). Nevertheless, data from both observational and interventional trials lack in healthy individuals free of cardiovascular disease—where long-term prevention efforts may be particularly effective.

We addressed these issues by investigating the associations of whole blood n-3 PUFA levels (given as the Omega-3 Index) with renal function and albuminuria in a large population of young healthy adults.

## Materials and Methods

### Study Population

For this cross-sectional study, baseline data from the GAPP study (genetic and phenotypic determinants of blood pressure and other cardiovascular risk factors) were analyzed. The GAPP study is an ongoing large population-based cohort study, which investigates the determinants of BP and other cardiovascular risk factors in young and healthy adults, described elsewhere (11). In brief, from 2010 to 2013 all inhabitants of the Principality of Liechtenstein in the given age range were invited and 2,170 healthy adults aged 25–41 years agreed to participate. Main exclusion criteria were established cardiovascular disease, chronic kidney disease, a BMI higher than 35 kg/m^2^, obstructive sleep apnea, daily intake of non-steroidal anti-inflammatory medication and diabetes.

From a total of 2,170 patients enrolled in the GAPP study, we excluded participants on ACE-Inhibitors (*n* = 5), angiotensin-receptor blockers (*n* = 22), diuretics (*n* = 9) as well as the data sets with missing information on ethnicity (*n* = 136), creatinine or cystatin C values (*n* = 9). Because of multiple exclusion criteria in one individual, 169 individuals were excluded, resulting in a total of 2,001 participants for this analysis.

The study protocol was approved by the local ethics committee (Cantonal Ethics Commission of Zurich, Zurich, Switzerland). Informed written consent was obtained from every individual participant.

### Blood Sampling and Whole Blood Fatty Acid Composition

At baseline, venous whole blood samples were collected from every participant after an overnight fast. The aliquoted cryotubes where immediately stored at −80°C (11), which has been shown to not alter the n-3 levels measured (12), and underwent no further freeze-thaw cycles. Whole blood fatty acids were analyzed according to the high sensitivity-Omega-3 Index methodology, initially described for erythrocyte samples (13). FA methyl esters were generated from whole blood by acid transesterification and analyzed by gas chromatography using a GC2010 Gas Chromatograph (Shimadzu, Duisburg, Germany) equipped with a SP2560, 100-m column (Supelco, Bellefonte, Pennsylvania, USA) hydrogen as carrier gas. FAs were identified by comparison with a standard mixture of FAs characteristic of erythrocytes. Results are given as relative amounts of EPA (C20:5n3) and DHA (C22:6n3), expressed as a percentage of a total of 26 identified FAs, referred to as Omega-3 Index. A validated correction factor was applied for whole blood aliquots (14, 15). Where mentioned, ALA (C18:3n3) is given as percentage of a total of 26 identified FAs. The coefficient of variation for FA levels was 5%. Analyses were quality controlled according to DIN ISO 15189.

### Assessment of Kidney Function, Albuminuria and Other Biomarkers

Plasma creatinine, Cystatin C and high-sensitivity C-reactive protein (hs-CRP) were assayed from fresh samples immediately after blood draw on a Roche Cobas analyzer (F. Hoffmann-La Roche, Basel, Switzerland). Estimated glomerular filtration rate (eGFR) given as *mL*/ min /1.73*m*^2^ was computed using the CKD-EPI Creatinine-Cystatin Equation (2012) as published by Inker et al. (16). Urine albumin and urine creatinine were obtained from fasting morning urine samples. Albuminuria was quantified with the ACR and given in mg/mmol. Hemoglobin A1c (HbA1c) was measured using HPLC.

### Other Study Variables

For all participants, a detailed assessment of personal, medical, lifestyle and nutritional factors was performed. Smoking status was self-assessed and categorized into current, past and never smokers. Moderate and vigorous physical activity was assessed using the validated international physical activity questionnaire (17). Regular physical activity was defined as moderate or vigorous physical activity of at least 75 or 150 min/week, respectively. Alcohol consumption and frequency of fruit, vegetable and fish consumption were obtained with the Swiss health survey questionnaire from 2007. Healthy diet was defined as at least five servings of fruits or vegetables per day. Highest educational status was reported. Weight and height were directly measured in a standardized way. BMI was calculated by dividing weight in kg by height in m^2^.

### Statistical Analysis

Data were tested against a predefined hypothesis, assuming an inverse association of Omega-3 Index with eGFR and ACR. Baseline characteristics were presented overall as well as stratified according to quartiles of Omega-3 Index in % (medians with upper and lower quartiles and p-values from Wilcoxon-Mann-Whitney or chi-squared tests). To assess the relationship of n-3 PUFAs with eGFR we fit simple and multivariable linear regression models with eGFR as the dependent variable.

To account for the skewed distribution and excess of zeros in ACR, we fit a generalized linear model using the Tweedie distribution and a log-link. Model coefficients were back-transformed by exponentiation and expressed as ratio. Six outlier observations with very high ACR (ranging from 79.77 to 104.22, see Supplementary Table 3) were excluded from the analysis as values were highly pathological and atypical for this set of healthy subjects.

For all fitted linear models, we assessed model assumptions (linearity, normality of residuals, and presence of influential values); all assumptions held true.

In addition, we evaluated the association of Omega-3, ALA, EPA and DHA with different eGFR quantiles to study the potential influence on people with high or low eGFR. We performed joint multivariate quantile regression using the method by Yang and Tokdar (18) implemented in the R package “qrjoint.” This exploratory analysis is presented in the Supplementary Material.

## Results

### Study Population

Baseline characteristics for the whole population and stratified by quartiles of the Omega-3 Index are shown in Table 1. Median age was 37 years (IQR 31; 40) and 53% were female. Median Omega-3 Index was 4.59 (IQR 4.06; 5.25). Median eGFR was 111 ml/min/1.73 m^2^ (IQR 103; 118). Median ACR was 0.14 mg/mmol (IQR 0; 0.43).

**Table 1:** Baseline characteristics.

	**All *n* = 2001**	**Quartile 1 *n* = 504**	**Quartile 2 *n* = 504**	**Quartile 3 *n* = 491**	**Quartile 4 *n* = 499**	***P*-value**
Omega-3 index range (%)	4.59 [4.06, 5.25]	2.43–4.06	4.08–4.59	4.60–5.25	5.26–9.26	
ALA (%)	0.23 [0.18,0.28]	0.22 [0.17,0.28]	0.23 [0.18,0.28]	0.23 [0.19,0.28]	0.24 [0.19,0.29]	
EPA (%)	0.62 [0.49, 0.78]	0.5 [0.41, 0.61]	0.57 [0.48, 0.68]	0.64 [0.53, 0.78]	0.8 [0.68, 1.06]	
DHA (%)	3.08 [2.62, 3.61]	2.37 [2.17, 2.54]	2.87 [2.73, 3.02]	3.34 [3.18, 3.5]	4.01 [3.78, 4.4]	
Sex (%)	m: 938 (47%), f: 1063 (53%).	m: 278 (55%), f: 226 (45%).	m: 240 (48%), f: 264 (52%).	m: 220 (45%), f: 271 (55%).	m: 198 (40%), f: 301 (60%).	<0.001
eGFR (ml/min)	110.88 [102.74, 118.27]	111.09 [102.79, 118.39]	110.32 [103,117.77]	110.47 [102.52, 117.78]	111.79 [102.5, 119.39]	0.60
ACR (mg/mmol)	0.14 [0, 0.43]	0.2 [0, 0.44]	0.19 [0, 0.46]	0 [0, 0.41]	0 [0, 0.37]	<0.01
ACR >3.0 mg/mmol	59 (3%),	19 (4%),	16 (3%),	13 (3%),	11 (2%),	0.50
Cystatin C Serum (mg/l)	0.79 [0.71, 0.87]	0.8 [0.72, 0.88]	0.79 [0.72, 0.87]	0.79 [0.72, 0.86]	0.76 [0.7, 0.85]	<0.001
Creatinine Serum (mg/dl)	0.76 [0.66, 0.87]	0.77 [0.66, 0.88]	0.77 [0.67, 0.87]	0.76 [0.66, 0.87]	0.76 [0.64, 0.87]	0.48
BMI (kg/m^2^)	24.02 [21.77, 26.85]	24.51 [22.14, 27.58]	24.42 [22.03, 27.05]	23.92 [21.85, 26.80]	23.34 [21.21, 25.90]	<0.001
Fruit/vegetable consumption (%)	no: 1,644 (82%), yes: 357 (18%)	no: 436 (87%), yes: 68 (13%)	no: 423 (84%), yes: 81 (16%)	no: 405 (82%), yes: 86 (18%)	no: 377 (76%), yes: 122 (24%)	<0.001
Smoking (%)	active: 430 (22%) never: 1,098 (55%) past: 471 (24%)	active: 163 (32%), never: 228 (45%), past: 112 (22%)	active: 125 (25%), never: 265 (53%), past: 113 (22%)	active: 89 (18%), never: 279 (57%), past: 123 (25%)	active: 53 (11%), never: 323 (65%), past: 123 (25%)	<0.001
Regular physical activity (%)	no: 373 (19%), yes: 1,620 (81%)	no: 88 (18%), yes: 411 (82%)	no: 93 (19%), yes: 409 (81%)	no: 97 (20%), yes: 393 (80%)	no: 94 (19%), yes: 405 (81%)	0.86
Education (%)	h: 766 (39%), l: 122 (6%), m: 1,092 (55%)	h: 135 (27%), l: 38 (8%), m: 321 (65%)	h: 185 (37%), l: 34 (7%), m: 281 (56%)	h: 194 (40%), l: 18 (4%), m: 273 (56%),	h: 250 (50%), l: 32 (6%), m: 216 (43%),	<0.001
24-h DBP (mmHg)	78.13 [73.08, 83.41]	79.09 [73.59, 85.15]	78.41 [73.41, 83.53]	77.84 [72.78, 82.55]	76.79 [72.22, 82.2]	<0.001
24-h SBP (mmHg)	122.56 [115.11, 130.71]	124.39 [116.54, 133.31]	123.06 [115.26, 130.58]	122.28 [115.18, 130.05]	120.3 [113.65, 128.35]	<0.001
Gly HbA1c (%)	5.4 [5.2, 5.6]	5.5 [5.2, 5.7]	5.4 [5.2, 5.6]	5.4 [5.2, 5.6]	5.4 [5.1, 5.6]	0.26
HDL (mmol/l)	1.5 [1.24, 1.79]	1.42 [1.17, 1.71]	1.49 [1.22, 1.76]	1.51 [1.24, 1.84]	1.58 [1.35, 1.86]	<0.001
LDL (mmol/l)	2.87 [2.35, 3.47]	2.98 [2.36, 3.6]	2.87 [2.38, 3.45]	2.88 [2.33, 3.47]	2.72 [2.28, 3.38]	0.05
Triglyceride (mmol/l)	0.84 [0.59,1.19]	0.98 [0.64,1.46]	0.87 [0.62, 1.21]	0.84 [0.59, 1.13]	0.75 [0.57, 1.02]	<0.001

*n = 2,001; Data are medians [interquartile range] or numbers (percentages). Regular physical activity = moderate (≥150 min per week) or vigorous physical activity (≥75 min per week). Fruit and vegetable consumption = fruit and vegetable consumption ≥5 servings per day. For education h = high, m = middle, l= low. BMI = body mass index, BP = blood pressure, f = female, h = hour, HDL = high density lipoprotein, hs-CRP = high-sensitivity C-reactive protein, LDL = low density lipoprotein, m = male*.

* *p-value was calculated using analysis of variance, Kruskal-Wallis test (due to asymmetry in most continuous variables) or chi-square test, as appropriate, in order to compare the respective values across the quartiles*.

Participants in the higher Omega-3 Index quartiles had significantly lower Cystatin C (*p* < 0.001). Further, a higher Omega-3was found in women than men and associated with a lower BMI, less smoking (*p*-value for all < 0.001) and a lower 24-h blood pressure profile (systolic *p* < 0.0001; diastolic *p* < 0.001). Additionally, individuals with a higher Omega-3 Index had higher HDL levels (*p* < 0.001), lower triglycerides (*p* < 0.001) and consumed significantly more fruits and vegetables (*p* < 0.001) as well as fish (*p* < 0.001). Groups with a higher Omega-3 Index also had a higher educational status (*p* < 0.001).

### Inverse Association of Omega-3 Index With ACR

Omega-3 Index was inversely associated with ACR before and after adjustment for potential confounders (Table 2). A 1-unit increase in the Omega-3 Index was associated with a 14% lower ACR (adjusted ratio 0.86, CI 0.74–1.00; *p* = 0.048). Analyses of individual fatty acids showed a significant association for DHA with a ratio of (0.82, CI 0.68–0.99, *p* = 0.037) but not for ALA (*p* = 0.69) or EPA (*p* = 0.40), as presented in Table 2.

**Table 2:** Crude and adjusted change in ACR for Omega-3 Index, ALA, EPA and DHA.

	**Crude ratio (95% CI)**	***P*-value**	**Adjusted ratio (95% CI)**	***P*-value**
Omega-3 Index (per unit [%])	0.84 (0.73–0.96)	**0.011**	0.86 (0.74–1.00)	**0.048**
ALA (per unit [%])	3.79 (0.97–14.76)	0.055	1.35 (0.30–6.03)	0.692
EPA (per unit [%])	0.70 (0.42–1.16)	0.165	0.80 (0.47–1.35)	0.401
DHA (per unit [%])	0.80 (0.67–0.95)	**0.011**	0.82 (0.68–0.99)	**0.037**

*Covariates used for adjustment are age, sex, BMI, smocking status, mean systolic blood pressure over 24 h, triglycerides, glycated hemoglobin A1c, high density lipoproteins*.

*Bold values represent that p < 0.05*.

### No Association of Omega-3 Index With eGFR

In linear regression analyses, no association between the Omega-3 Index and eGFR was found (Table 3); the adjusted difference in eGFR was −0.21 ml/min (95%-CI −0.76;0.35; *p* = 0.466) per 1-unit increase in Omega-3 Index. Additional analyses for individual fatty acids (ALA, EPA and DHA) also showed no significant association with eGFR (Table 3). Moreover, no significant association was found in non-linear additive models (Supplementary Material).

**Table 3:** Crude and adjusted difference in eGFR for Omega-3 Index, ALA, EPA, and DHA.

	**Crude difference (95% CI)**	***P*-value**	**Adjusted difference (95% CI)**	***P*-value**
Omega-3 Index (per unit [%])	−0.07 (−0.61 to 0.46)	0.794	−0.21 (−0.76 to 0.35)	0.466
ALA (per unit [%])	−1.81 (−7.46 to 3.84)	0.530	−0.72 (−6.58 to 5.15)	0.811
EPA (per unit [%])	0.70 (−1.23 to 2.63)	0.476	1.49 (−0.51 to 3.49)	0.144
DHA (per unit [%])	−0.20 (−0.88 to 0.48)	0.571	−0.50 (−1.21 to 0.20)	0.162

*Covariates used for adjustment were age, sex, BMI, smoking status, mean systolic blood pressure over 24 h, triglycerides, glycated hemoglobin A1c and high-density lipoproteins*.

## Discussion

Our main findings collectively indicate that n-3 FA intake, as reliably reflected by the Omega-3 Index, is inversely associated with ACR in young and healthy individuals with preserved eGFR. Thus, Omega-3 fatty acids may exhibit cardio- and nephroprotective effects in healthy individuals through modulation of the ACR.

Albuminuria is a well-established early marker not only for kidney damage but also as a cardiovascular risk factor. Both prospective and epidemiologic data support albuminuria as a predictor for cardiovascular morbidity and mortality as well as all-cause mortality (6)—not only in individuals already suffering from hypertension or diabetes, but also in the general population. Even a minimal increase in ACR is associated with an increased risk of kidney failure and cardiovascular disease (19). Moreover, an increased ACR predicts incident hypertension and cardiovascular disease mortality at an 11-year follow-up, highlighting its potential as a target for preventive interventions (20).

In CKD treatment, the reduction of albuminuria remains a central pillar. Concerning the effects of n-3 PUFAs, longitudinal, interventional studies focusing on healthy adults with preserved kidney function, where prevention measures could be of particular importance are not available (21). From an interventional perspective, Elajami et al. (10) showed, that EPA and DHA supplementation was able to attenuate the progression of established albuminuria in patients with type 2 diabetes mellitus and coronary artery disease, most of whom were already under treatment with angiotensin-converting enzyme inhibitors or angiotensin-receptor blockers.

There is increasing evidence, that Omega-3 fatty acids are crucial in resolving inflammatory responses as substrates for the endogenic production of specialized pro-resolving mediators (resolvins, protectins, and maresins)—stopping inflammation and giving way to tissue reparation (22, 23). Priante et al. (24) provided a number of mechanism how n-3 PUFAs counteract renal inflammation and fibrosis. Further, n-3 PUFA supplementation affects cell junctions and protects from distal tubular cell damage in rat kidneys, thus directly and indirectly affecting albuminuria (25, 26). There is increasing evidence for differing individual effects of EPA and DHA on cardiometabolic risk factors (27). In our collective, the inverse association of n-3 PUFAs with ACR was mainly driven by DHA. Accordingly, several mechanistic studies provided evidence for DHA suppressing inflammation pathways in the kidney—of note, oxygenated metabolites were decreased more effectively by DHA than EPA (28–30).

The observed inverse association of Omega-3 fatty acids with ACR is concordant with a multitude of well-established beneficial effects of n-3 fatty acids on the cardiovascular system (31). Hypertension, oxidative stress, dyslipidemia, endothelial dysfunction and thrombogenesis are all intermediaries in the pathogenesis of both kidney disorders and cardiovascular disease—and all are positively modifiable through n-3 PUFAs (21, 32, 33). As already shown earlier by our group, omega-3 fatty acids are inversely associated with blood pressure in the same, mostly normotensive population, which may be a driver for kidney damage in young and healthy individuals (4). Further, participants with more favorable fatty acid profile at baseline have significantly lower triglyceride and higher HDL levels. The decreased risk of ischemic events through n-3 PUFA mediated triglyceride level modification was impressively demonstrated in the REDUCE-IT trial, where very high dosages of n-3 PUFAS were administrated (34). In contrast, the median Omega-3 Index of 4.6% in our population is highly comparable to data from other Western countries with an average Index of 4–5% (35–37), but still well-below the 8% recommended for optimal cardioprotection (14) or countries with high fish consumption like Japan (Omega-3 Index between 8 and 10%) (38). This suggests, that in the measured range of our population, no specific lower n-3 threshold exists. As demonstrated in the JELIS trial for major coronary event, n-3 PUFA is likely to be a continuous beneficial variable, where no ceiling effect was observed (39). Both, more favorable lipid profiles as well as lower blood pressure might mediate the observed effects. Further, participants with a higher Omega-3 Index are better educated, eat healthier and do more exercise. Nevertheless, our results remained significant also after adjustment for potential confounders.

Concerning the effect of n-3 PUFAs on eGFR, Miller et al. (40) suggest in their metanalysis that n-3 PUFA supplementation reduces urine protein excretion but not decline in GFR. While also in our population no association of Omega-3 Index with eGFR could be found, one should consider several factors when interpreting these results: First, the CKD-EPI formula, as all other formulas calculating the glomerular filtration rate, remains an estimation. Especially in patients with preserved eGFR, this estimation becomes less accurate and may not detect fine differences between individuals (41). Further, a quantifiable reduction in eGFR is a sign of significant and advanced kidney damage—not to be expected in this cohort of generally young and healthy adults. However, Cystatin C as a probably superior predictor of GFR in individuals with normal and mildly impaired kidney function (42) significantly differed across Omega-3 Index quartiles at baseline. This could also be explained with effects, in contrast to ACR findings, only grasping with higher n-3 PUFA levels—reflected in the drop of Cystatin C in the highest Omega-3 Index quartile. Interestingly, an analysis of the serum n-3 PUFA, creatinine and Cystatin C levels in 549 Japanese community-dwellers by Higashiyama et al. (43) found an association of n-3 PUFA fatty acids with Cystatin C based but not creatinine based eGFR estimation. Whether higher Omega-3 fatty acid levels may have a preventive effect on physiologic (accelerated) decline in kidney function is to be determined in longitudinal studies.

The main strengths of our study include the availability of both creatinine and cystatin C for the computation of the eGFR, which permitted us to implement the internationally recognized combined CKD-EPI formula (16). Additionally, we utilized the still novel Omega-3 Index as a marker for n-3 PUFA intake. This method is proven to have a reduced biologic and analytic variability compared to plasma levels and thus reliably reflects and individual's long-term omega-3 status and tissue content (12). It is robust and resistant to short-term variation of n-3 PUFA intake and obviously superior to often unreliable nutritional questionnaires (13). Together with definition and homogeneity of our large and well-characterized study cohort composed of the young and healthy population of Liechtenstein with access to state-of-the-art preventive and medical service, it allows comparability to other Western societies. Further, we were careful to exclude individuals with known renal insufficiency, on ARBs as well as ACEs or suffering from any major illness including diabetes—thus allowing to minimize residual confounding.

Limitations include the cross-sectional design not allowing to infer causal relationship or to determine the directionality of the observed effects. Also, residual confounding despite extensive adjustment cannot be completely ruled out. Our findings are also limited to the observed population (white, affluent young and healthy) with its specific characteristics including usually healthy nutritional habits. Further, the use of single spot-urine after an overnight fast, rather than repeated measurements or the gold-standard of 24-h collection may have influenced our results for albuminuria. Fourth, none of the results were adjusted for multiple testing, given a clear, predefined hypothesis and to avoid potential over-adjustment because of significant correlations between individual fatty acids. Lastly, we do not have data on the source of n-3 PUFAs or the number of individuals taking supplements. The latter is likely to be very low given the relatively low levels of the n-3 concentrations measured, as also shown in a study by Shen et al. (44) linking measured Omega-3 Index levels to self-reported fatty acid intake. Overall, detailed information on the n-3 PUFA source is probably not of relevance to the association, with the Omega-3 Index representing an active biologic analog (independent of data on the form and frequency of intake).

In conclusion, a higher Omega-3 Index was significantly associated with a lower ACR in this cohort of young healthy individuals with preserved GFR. In addition to cardiovascular preventive properties, Omega-3 fatty acids may have a protective effect on early kidney injury. The supplementation and/or higher intake of n-3 PUFAs may provide a well-tolerated, cost-effective approach for the prevention of chronic kidney disease—another established cardiovascular risk factor with its own substantial burden of disease and socioeconomic footprint.

## Data Availability Statement

The raw data supporting the conclusions of this article will be made available by the authors, without undue reservation.

## Ethics Statement

The studies involving human participants were reviewed and approved by Cantonal Ethics Commission of Zurich, Zurich, Switzerland. The patients/participants provided their written informed consent to participate in this study.

## Author Contributions

MF, DC, and JB contributed to the conception or design of the work. All authors contributed to the acquisition, analysis, or interpretation of data for the work. MF drafted the manuscript. SA, SR, MRe, DC, and JB critically revised the manuscript. All gave final approval and agree to be accountable for all aspects of work ensuring integrity and accuracy.

## Conflict of Interest

JB has received research and educational grants and honoraria from Amgen, Bayer HealthCare, Boehringer Ingelheim, Daiichi Sankyo, MARS, Inc. and Novartis outside this study. DC received honoraria from Servier, Canada, outside of this study. The remaining authors declare that the research was conducted in the absence of any commercial or financial relationships that could be construed as a potential conflict of interest.
