# Supplementary material for: Blood Omega-3 Fatty Acids Are Inversely Associated With Albumin-Creatinine Ratio in Young and Healthy Adults (The Omega-Kid Study)
Source: Front Cardiovasc Med. 2021 Apr 27;8:622619. doi: 10.3389/fcvm.2021.622619 (PMC8110728; doi:10.3389/fcvm.2021.622619)
Supplement: Supplementary file 1 [file Data_Sheet_1.docx]

**Supplemental material: Blood omega-3 fatty acids are inversely associated with albumin-creatinine ratio in young and healthy adults (The Omega-Kid Study)**

**Table S01.** Crude and adjusted associations of eGFR quantiles with Omega-3 Index, ALA, EPA and DHA.

|  | **eGFR quantile** | **Crude effect (95% CI)** | **Adjusted effect (95% CI)** |
| --- | --- | --- | --- |
| **Omega-3 Index** | 0.1 | -0.790 (-1.802 to -0.071) | -0.825 (-1.685 to -0.120) |
|  | 0.2 | -0.406 (-1.135 to 0.282) | -0.609 (-1.392 to -0.040) |
|  | 0.3 | -0.228 (-1.018 to 0.495) | -0.465 (-1.111 to 0.070) |
|  | 0.4 | -0.099 (-0.808 to 0.552) | -0.331 (-1.098 to 0.115) |
|  | 0.5 | 0.009 (-0.648 to 0.644) | -0.236 (-1.072 to 0.204) |
|  | 0.6 | 0.142 (-0.363 to 0.775) | -0.102 (-0.817 to 0.451) |
|  | 0.7 | 0.259 (-0.242 to 0.836) | 0.080 (-0.491 to 0.581) |
|  | 0.8 | 0.387 (-0.259 to 0.917) | 0.145 (-0.489 to 0.721) |
|  | 0.9 | 0.507 (-0.239 to 1.232) | 0.202 (-0.510 to 1.034) |
| **ALA** | 0.1 | -2.079 (-12.060 to 2.946) | -2.421 (-8.522 to 4.976) |
|  | 0.2 | -1.527 (-10.684 to 4.048) | -2.247 (-7.683 to 5.455) |
|  | 0.3 | -1.502 (-8.691 to 3.776) | -2.316 (-7.695 to 5.729) |
|  | 0.4 | -1.827 (-6.862 to 3.624) | -2.490 (-6.560 to 5.692) |
|  | 0.5 | -2.072 (-6.485 to 3.055) | -2.126 (-6.615 to 5.364) |
|  | 0.6 | -1.854 (-6.145 to 2.977) | -1.834 (-6.273 to 5.191) |
|  | 0.7 | -1.818 (-5.703 to 3.478) | -1.554 (-5.843 to 4.983) |
|  | 0.8 | -2.176 (-6.302 to 4.634) | -1.536 (-6.063 to 4.774) |
|  | 0.9 | -2.640 (-7.570 to 3.446) | -1.489 (-6.324 to 4.865) |
| **EPA** | 0.1 | 1.186 (-1.611 to 4.453) | 1.048 (-0.972 to 4.032) |
|  | 0.2 | 1.765 (-1.235 to 5.031) | 1.694 (0.073 to 3.068) |
|  | 0.3 | 1.499 (-1.473 to 4.436) | 1.428 (-0.150 to 3.024) |
|  | 0.4 | 1.231 (-1.766 to 4.206) | 1.238 (-0.833 to 2.755) |
|  | 0.5 | 1.174 (-1.827 to 3.899) | 1.512 (-0.118 to 3.233) |
|  | 0.6 | 1.223 (-1.516 to 3.489) | 2.120 (0.695 to 3.486) |
|  | 0.7 | 1.351 (-1.155 to 3.394) | 2.410 (1.090 to 4.011) |
|  | 0.8 | 1.433 (-0.799 to 3.358) | 2.649 (1.038 to 4.526) |
|  | 0.9 | 1.639 (-0.869 to 4.143) | 2.863 (0.901 to 4.883) |
| **DHA** | 0.1 | -0.998 (-1.939 to -0.078) | -1.171 (-2.228 to -0.296) |
|  | 0.2 | -0.428 (-1.337 to 0.502) | -0.896 (-1.581 to -0.305) |
|  | 0.3 | -0.069 (-1.008 to 1.011) | -0.691 (-1.423 to -0.145) |
|  | 0.4 | 0.149 (-0.751 to 1.188) | -0.579 (-1.294 to 0.019) |
|  | 0.5 | 0.276 (-0.609 to 1.171) | -0.507 (-1.154 to 0.127) |
|  | 0.6 | 0.341 (-0.475 to 1.072) | -0.429 (-1.078 to 0.209) |
|  | 0.7 | 0.306 (-0.476 to 0.953) | -0.333 (-1.002 to 0.365) |
|  | 0.8 | 0.252 (-0.468 to 0.926) | -0.198 (-0.973 to 0.567) |
|  | 0.9 | 0.235 (-0.362 to 0.965) | -0.141 (-0.975 to 0.946) |

‘95 CI’ stands for 95% credible interval. Covariates used for adjustment were age, sex, BMI, smoking status, mean systolic blood pressure over 24 hours, triglycerides, glycated hemoglobin A1c and high-density lipoproteins.

**Figure S02.** Illustration to Table S01 for crude and adjusted associations of eGFR quantiles with Omega-3 Index, ALA, EPA and DHA.


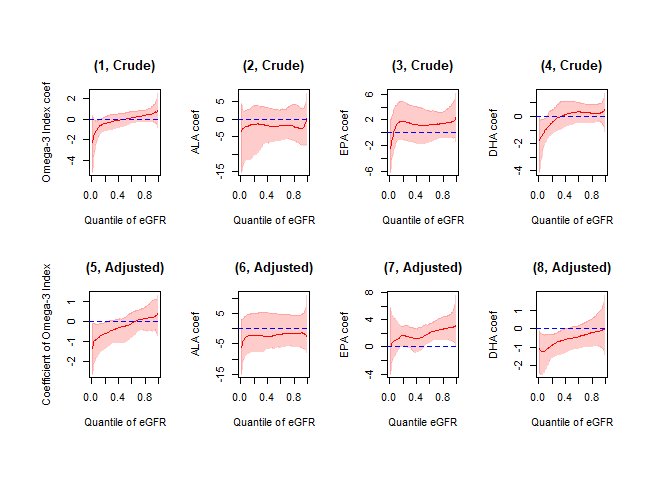


**Table S03.** Observations removed from Tweedie generalized linear and additive models as too influential.

| **ACR (mg/mmol)** | **eGFR** |
| --- | --- |
| 79.77387 | 104.04449 |
| 38.84786 | 110.50153 |
| 44.26270 | 115.93342 |
| 65.61303 | 105.04179 |
| 127.93656 | 84.05781 |
| 104.22134 | 116.16104 |

**Table S04.** Logistic regression estimates for albuminuria.

|  |  | **Crude odds ratio (95% CI)** | **P-value** | **Adjusted odds ratio (95% CI)** | **P-value** |
| --- | --- | --- | --- | --- | --- |
| **Omega-3 Index** | continuous (per %) | 0.80 (0.60 to  1.08) | 0.151 | 0.80 (0.58 to  1.10) | 0.170 |
|  | 1st quartile | Reference |  | Reference |  |
|  | 2nd quartile | 0.84 (0.43 to  1.65) | 0.606 | 0.82 (0.40 to  1.67) | 0.584 |
|  | 3rd quartile | 0.69 (0.34 to  1.42) | 0.318 | 0.65 (0.30 to  1.40) | 0.272 |
|  | 4th quartile | 0.58 (0.27 to  1.22) | 0.150 | 0.58 (0.26 to  1.28) | 0.177 |
| **ALA** | continuous (per %) | 17.32 (1.58 to  189.54) | 0.019 | 5.95 (0.40 to  88.18) | 0.195 |
|  | 1st quartile | Reference |  | Reference |  |
|  | 2nd quartile | 1.54 (0.69 to  3.44) | 0.286 | 1.34 (0.59 to  3.03) | 0.489 |
|  | 3rd quartile | 1.50 (0.65 to  3.46) | 0.339 | 1.26 (0.54 to  2.95) | 0.587 |
|  | 4th quartile | 2.13 (0.99 to  4.60) | 0.054 | 1.55 (0.69 to  3.47) | 0.288 |
| **EPA** | continuous (per %) | 0.81 (0.29 to  2.25) | 0.681 | 1.12 (0.40 to  3.18) | 0.828 |
|  | 1st quartile | Reference |  | Reference |  |
|  | 2nd quartile | 1.16 (0.60 to  2.26) | 0.664 | 1.33 (0.66 to  2.66) | 0.429 |
|  | 3rd quartile | 0.50 (0.21 to  1.16) | 0.106 | 0.62 (0.26 to  1.50) | 0.288 |
|  | 4th quartile | 0.94 (0.46 to  1.90) | 0.855 | 1.27 (0.58 to  2.77) | 0.545 |
| **DHA** | continuous (per %) | 0.74 (0.51 to  1.07) | 0.113 | 0.70 (0.47 to  1.05) | 0.085 |
|  | 1st quartile | Reference |  | Reference |  |
|  | 2nd quartile | 0.85 (0.44 to  1.65) | 0.633 | 0.92 (0.46 to  1.84) | 0.819 |
|  | 3rd quartile | 0.54 (0.26 to  1.15) | 0.110 | 0.49 (0.22 to  1.10) | 0.085 |
|  | 4th quartile | 0.55 (0.26 to  1.15) | 0.113 | 0.53 (0.24 to  1.18) | 0.122 |

Odds ratios for albuminuria presented with crude and covariates adjusted logistic regression models with logit link. Covariates used for adjustment were: age, sex, BMI, smocking status, mean systolic blood pressure over 24 hours, triglycerides, glycated hemoglobin A1c, high density lipoproteins.
